# Transcutaneous auricular vagus nerve stimulation alters cough sensitivity depending on stimulation parameters: potential implications for aspiration risk

**DOI:** 10.3389/fnins.2024.1265894

**Published:** 2024-02-08

**Authors:** Karen B. Ng, Esther Guiu Hernandez, Jillian Haszard, Phoebe Macrae, Maggie-Lee Huckabee, Yusuf O. Cakmak

**Affiliations:** ^1^Department of Anatomy, School of Biomedical Sciences, University of Otago, Dunedin, New Zealand; ^2^University of Canterbury Rose Centre for Stroke Recovery and Research, Christchurch, New Zealand; ^3^Division of Health Sciences, Biostatistics Centre, University of Otago, Dunedin, New Zealand; ^4^School of Psychology, Speech and Hearing, University of Canterbury, Christchurch, New Zealand; ^5^Point-of-Care Technologies Theme, Centre for Bioengineering, University of Otago, Dunedin, New Zealand

**Keywords:** cough, cough sensitivity, citric acid, transcutaneous auricular vagus nerve stimulation, frequency, stimulation parameters

## Abstract

**Background:**

Transcutaneous auricular vagus nerve stimulation (taVNS) is considered a safe and promising tool for limb rehabilitation after stroke, but its effect on cough has never been studied. It is known that the ear and larynx share vagal afferent pathways, suggesting that stimulating the ear with taVNS might have effects on cough sensitivity. The specific stimulation parameters used can influence outcomes.

**Objective:**

To investigate the effect of various stimulation parameters on change in cough sensitivity, compared to the reference parameter of 25 Hz stimulation at the left concha (most commonly-used parameter for stroke rehabilitation). Design, setting, and participants: Randomized, single-blind, active-controlled, eight-period cross-over design conducted March to August 2022 at a New Zealand research laboratory with 16 healthy participants.

**Interventions:**

All participants underwent eight stimulation conditions which varied by stimulation side (right ear, left ear), zone (ear canal, concha), and frequency (25 Hz, 80 Hz). Main outcome measures: Change in natural and suppressed cough threshold (from baseline to after 10 min of stimulation) assessed using a citric acid cough reflex test.

**Results:**

When compared to the reference parameter of 25 Hz stimulation at the left concha, there was a reduction in natural cough threshold of −0.16 mol/L for 80 Hz stimulation at the left canal (*p* = 0.004), indicating increased sensitivity. For the outcome measure of suppressed cough threshold, there was no significant effect of any of the stimulation conditions compared to the active reference.

**Conclusion:**

Since stroke patients often have cough hyposensitivity with resulting high risk of silent aspiration, using 80 Hz taVNS at the left canal may be a better choice for future stroke rehabilitation studies than the commonly used 25 Hz taVNS at the left concha. Treatment parameters should be manipulated in future sham-controlled trials to maximize any potential treatment effect of taVNS in modulating cough sensitivity.

**Clinical trial registration:**

ACTRN12623000128695.

## 1 Introduction

Transcutaneous auricular vagus nerve stimulation (taVNS) is a type of non-invasive neuromodulation that has recently received increasing interest as a promising tool for sensory and motor rehabilitation after stroke (Xie et al., 2021; Baig et al., 2022; Li et al., 2022). A search of “transcutaneous auricular vagus nerve stimulation AND stroke” on PubMed reveals that the number of research publications on this topic has steadily increased from 2015, more than doubling by the year 2022. taVNS has been claimed to improve aspects of sensory and motor functioning after stroke, including upper limb sensation (Baig et al., 2019), limb movement (Redgrave et al., 2018b; Wu et al., 2020), and swallowing (Wang et al., 2022).

taVNS provides surface electrical stimulation to the afferent nerves of the ear. It is generally considered safe and non-invasive (Redgrave et al., 2018a), however possible effects on cough function should be considered. Vagal afferent fibers which emerge at the cutaneous surface of the ear also innervate the laryngeal mucosa that regulates cough and laryngeal sensation. The ear and larynx have shared sensory innervation pathways from the vagus nerve. This is demonstrated by the phenomenon of referred ear pain, where pain caused by pharyngeal and laryngeal cancers are projected along the laryngeal branch of the vagus nerve to the territory of the auricular branch of the vagus nerve, and subjectively experienced in the ear canal (Weissman, 1997). It is important to note that stimulation of the external auditory canal, for example during medical examination of the ear or as a result of impacted ear wax, can elicit coughing (also called Arnold’s ear-cough reflex; Jegoux et al., 2002; Gold et al., 2017). Conversely, removal of the ear wax can alleviate chronic cough (Jegoux et al., 2002). These well-documented reciprocal interactions between the auricular skin and laryngeal mucosa suggest there may be unintended effects on cough function when taVNS is applied.

After implanted VNS for epilepsy, 66% of participants have laryngopharyngeal dysfunction (including the presence of coughing; Giordano et al., 2017). The adverse events from non-invasive taVNS are less frequently reported. Although symptoms of pharyngeal and laryngeal pain have been noted (Bauer et al., 2016; Gaul et al., 2016; Redgrave et al., 2018a), no study has investigated the potential change in cough threshold with taVNS, and whether these changes are parameter-specific. Subtle changes in laryngeal sensitivity may not be picked up through patient report and may only be identified if cough sensitivity is systematically and objectively measured before and after stimulation.

It is important to understand the potential risks of taVNS on cough function, since stroke patients are likely to have blunted cough reflex due to neurological damage from stroke (Kobayashi et al., 1994; Vilardell et al., 2017). If taVNS increases the cough threshold, using taVNS as stroke rehabilitation may further depress the cough reflex or halt the recovery process. Previous research has demonstrated that reduced cough response to a cough reflex test is significantly associated with a higher risk of silent aspiration (foreign material entering the airway without a cough response or other clinical indication) and aspiration pneumonia (Addington et al., 1999, 2005; Miles et al., 2013; Nakashima et al., 2018). On the other hand, if taVNS decreases the cough threshold, people with chronic cough who have laryngeal hypersensitivity may be at risk of increased, excessive coughing due to a heightened cough reflex (Ryan et al., 2014; Dicpinigaitis et al., 2018; Mai et al., 2020). However, the modulation of cough thresholds in both directions could be used as treatment, either to enhance laryngeal sensitivity in individuals with absent cough reflex, or reduce hypersensitivity in those with chronic cough. In this context, it is important for clinicians to be aware of the potential effects of taVNS on cough sensitivity to ensure safety and understand potential treatment effects.

Specific taVNS parameters that can influence functional outcomes include stimulation frequency and stimulation location (Badran et al., 2018; Thompson et al., 2021). In the stroke rehabilitation literature, the most commonly used stimulation parameters are 20–25 Hz frequency and left concha location (Butt et al., 2020; Thompson et al., 2021). However, previous research suggests that high frequency (80 Hz) auricular stimulation is more effective at improving sensory (olfactory) performance compared to low frequency (Maharjan et al., 2018). In addition, the ear canal is densely populated with auricular branch of vagus nerve fibers (Wadie et al., 2013; Watanabe et al., 2016), explaining the presence of referred pain in the ear canal with laryngeal cancers (Weissman, 1997; Norris and Koontz, 2020). Multiple studies have demonstrated that mechanically stimulating the ear canal evokes the cough reflex (Jegoux et al., 2002; Gold et al., 2017), suggesting that the ear canal may be a promising location for application of taVNS. Manipulating stimulation frequency and location can have significant effects on motor and sensory functioning, and it is unknown whether the standard parameters of 25 Hz taVNS on the left concha is optimal for modulating cough sensitivity.

Most studies use the left ear for non-invasive taVNS, based on the protocol for invasive VNS where right ear stimulation is contraindicated due to safety concerns. However, a systematic review found that cardiac side effects from taVNS are rare, regardless of the side of stimulation (Redgrave et al., 2018a). Vagal innervation of the laryngeal mucosa is largely ipsilateral, although innervation may not be symmetrical, and lateral dominance cannot be excluded (Wadie et al., 2013). It’s therefore possible that cough sensitivity is differentially affected by right vs. left stimulation.

This study measured cough reflex thresholds before and after 10 min of taVNS in healthy participants. There were eight different stimulation conditions, including the most common parameter used for stroke rehabilitation research (25 Hz stimulation at the left concha). The seven stimulation conditions were compared to 25 Hz stimulation at the left concha in order to determine the potential effect of condition on modulating cough threshold. Results of this study will shed light on whether current standard stimulation parameters or an alternative should be used in future patient studies investigating cough rehabilitation in stroke.

## 2 Materials and methods

Data collection was undertaken at the University of Canterbury Rose Center for Stroke Recovery and Research. All participants provided written informed consent. Ethical approval for this study was obtained from the University of Otago Human Ethics (Health) Committee, reference number H21/106. The study was registered on the Australia New Zealand Clinical Trials Registry (Trial ID: ACTRN12623000128695).

### 2.1 Participants

Sixteen healthy adults (15 female, age: mean ± SD = 30 ± 10.7 years, range = 21–53 years) met inclusion/exclusion criteria and were enrolled in the study. Exclusionary criteria included: a history of swallowing, psychiatric, neurological, gastro-esophageal reflux, or cardio-respiratory disorders (e.g., chronic obstructive pulmonary disease or asthma); a recent (<2 weeks) acute upper respiratory tract infection; current or previous smoking; currently taking opioid or angiotensin-converting enzyme (ACE) inhibiting drugs; pregnant; an implanted device (e.g., pacemaker). These factors may affect cough sensitivity and/or have safety concerns when using taVNS.

A previous sensory rehabilitation study indicated cough threshold SD of 0.7 mol/L (Wallace et al., 2019). Using this and a conservative within-person correlation of 0.5, *n* = 13 participants were needed to detect a difference of 0.6 mol/L with 80% power to the alpha = 0.05 level. To allow for 20% drop out and a balanced design, 16 participants were recruited.

### 2.2 Study design

**Figure 1:**
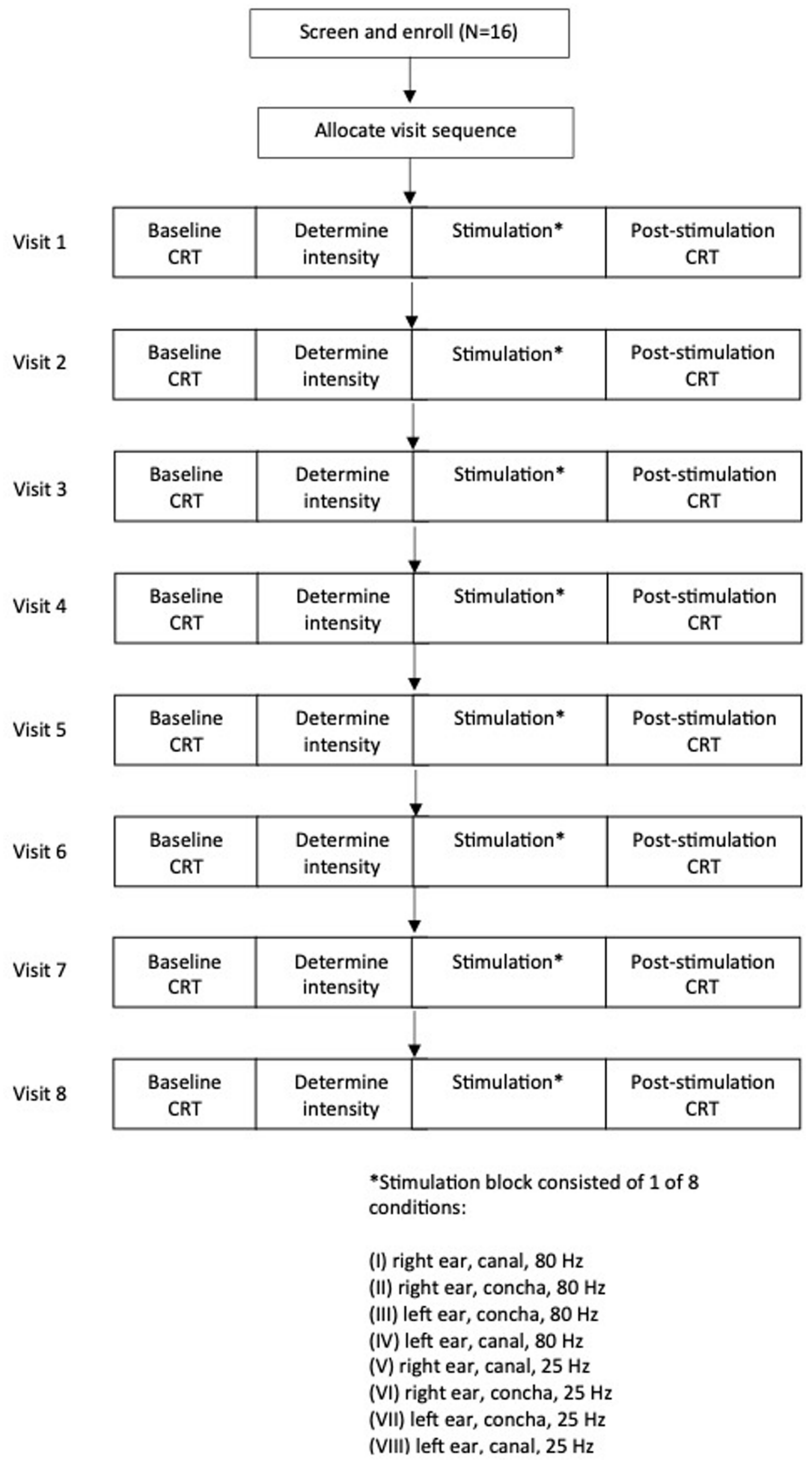
Participant flow diagram demonstrating study design and timeline of experimental visits. CRT, cough reflex test.

A randomized single-blind, active-controlled, cross-over design was used where participants completed all eight conditions. Stimulation parameters varied by stimulation side (right ear, left ear), stimulation zone (ear canal, concha), and stimulation frequency (25 Hz, 80 Hz), for a total of eight combinations of parameters: (I) right, canal, 80 Hz, (II) right, concha, 80 Hz, (III) left, concha, 80 Hz, (IV) left, canal, 80 Hz, (V) right, canal, 25 Hz, (VI) right, concha, 25 Hz, (VII) left, concha, 25 Hz, (VIII) left, canal, 25 Hz (Figure 1). The primary outcome measures were change in natural and suppressed cough thresholds, from baseline (immediately before stimulation) to post-stimulation (immediately after stimulation).

Each participant underwent one condition per visit, for a total of eight 1-h visits. Order of visits were incompletely counterbalanced between participants. Two balanced Latin squares (Masson, 2023) were used to generate 16 different visit sequences, and participants were randomly assigned to a sequence. There were at least 2 days washout period between visits to mitigate crossover of stimulation effects. The washout period duration was based on previous auricular stimulation research that found that there were significant improvements in gait at 20 and 40 min after auricular stimulation, but these gains regressed and were not significantly different to baseline by 60 min after the initial stimulation (Cakmak et al., 2020). Therefore, for our study, a washout period of at least 2 days was considered adequate for the participant’s cough sensitivity to fully return to baseline before the next stimulation visit. Actual washout period duration ranged from 2–82 days between sessions, with a right-skewed distribution (median: 5 days, interquartile range: 4 days). Variation in washout period was due to participant availability in scheduling the 8 sessions.

Study design and timeline for each visit is shown in Figure 1. Participants were blinded to the visit sequence.

### 2.3 Transcutaneous auricular vagus nerve stimulation

**Figure 2:**
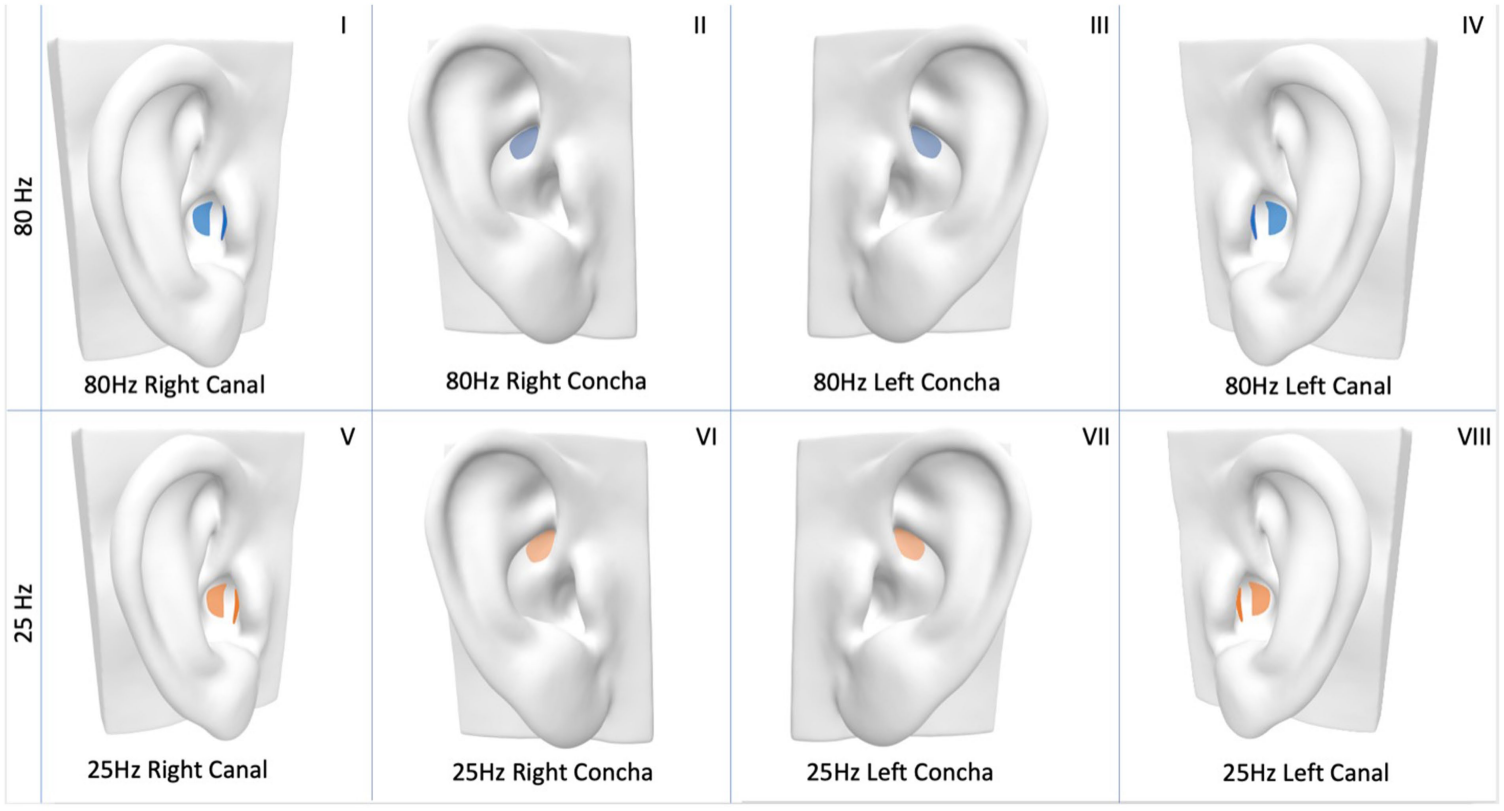
Electrode placement for transcutaneous auricular vagus nerve stimulation (taVNS). Concha placement—one electrode on concha, a second electrode on corresponding posterior surface of the ear (not shown). External auditory canal stimulation—one electrode on anterior wall, and another on the posterior wall of ear canal. Eight different taVNS stimulation parameters are demonstrated: (I) 80 Hz, right, canal, (II) 80 Hz, right, concha, (III) 80 Hz, left, concha, (IV) 80 Hz, left, canal, (V) 25 Hz, right, canal, (VI) 25 Hz, right, concha, (VII) 25 Hz, left, concha, (VIII) 25 Hz., left, canal.

taVNS was delivered to the seated participant for 10 min using a custom-built electrical signal generator. Pulse width for all conditions was set at 60 μs. The participant’s ear was inspected for any open sores, cuts, or lesions, and any jewelry was removed. The surface of the skin was cleaned with an alcohol wipe to ensure adequate conductivity. Single-use flexible hydrogel electrodes (RELI-Stick, Soterix Medical, New York, NY, United States) were placed on the ear in one of two locations (Figure 2), with electrode gel applied on the electrodes. For the external auditory canal location, two electrodes were attached to a foam earplug using double-sided adhesive tape. The earplug was compressed and then inserted into the ear canal, with one electrode making contact with the anterior wall and the other with the posterior wall of the ear canal following re-expansion. Previous studies have shown that Arnold’s ear-cough reflex was mostly elicited by palpating the posterior-inferior wall of the external auditory canal, but the anterior-inferior wall also elicited a response in a minority of participants (Tekdemir et al., 1998). For the concha location, one electrode was adhered to the skin of the cymba concha and a second electrode on the corresponding posterior surface of the external ear, and held in place with surgical tape.

Stimulation intensity was determined individually for each participant, as the stimulus intensity of taVNS needed to activate the afferent fibers of the vagus nerve differs between individuals (Ellrich, 2011). Once the electrodes were in place on the ear, stimulation intensity was adjusted at the beginning of each stimulation block as above the participant’s detection threshold and below their pain threshold. This was done by providing stimulation to the target area, starting from 1 mA and then gradually increasing stimulation intensity in 1 mA increments. Stimulation intensity was increased and decreased as needed until participant provided verbal confirmation that the intensity was high enough to subjectively elicit a “tingling” sensation, but just below an intensity that produced an unpleasant prickling sensation (Ellrich, 2011).

### 2.4 Outcome measures

Citric acid cough reflex testing was used to measure changes in cough sensitivity, immediately before and after stimulation. Citric acid was dissolved in sterile physiologic saline (0.9% sodium chloride) and was delivered through a mouthpiece attached to a jet nebulizer (DeVilbiss 646) using a breath-activated dosimeter (Koko Dosimeter) driven by an air compressor (Pulmo-Aide Compressor Nebulizer 5650I). The nebulizer was fitted with an inspiratory flow regulator valve (RIFR, nSpire Health Inc.) which limited inspiratory flow to 0.5 L/s. The orientation of the impinger arm and the distance between the capillary tube and jet orifice was kept constant within and across participants to ensure consistency of nebulizer output (Crapo, 2000). Prior to data collection, volume and reliability of nebulizer output was tested to ensure reproducibility between tests.

Participants were seated upright for the cough reflex test, with a nose clip in place. They inhaled linearly increasing concentrations of citric acid (0.01, 0.1, 0.3, 0.5, 0.7, 0.9, 1.1, 1.3, 1.5, 1.7 mol/L) through a mouthpiece. Placebo doses of saline were interspersed at specific points consistent across participants. For each trial, three 1.2 s doses of citric acid or saline were inhaled, with a minimum of 30-s break between trials. Citric acid and saline were removed from the refrigerator at least 45 min before data collection to allow the solutions to warm to room temperature.

Cough sensitivity threshold was defined as the lowest concentration at which 2 consecutive coughs (C2 response) were elicited during inhalation of the mists and up to 3 s after the third inhalation. Both natural cough threshold (NCT) and suppressed cough threshold (SCT) were tested. NCT was established first, by asking participants to “inhale and exhale three times, and cough if you need to.” SCT was then determined by beginning testing at one increment of citric acid concentration below the NCT (as SCT is typically higher than the NCT), and instructing participants to “inhale and exhale three times, and try not to cough.” Instructing participants to suppress their cough may provide a more accurate measure of laryngeal sensitivity, as the NCT is susceptible to the placebo effect (Hegland et al., 2012; Monroe et al., 2014).

### 2.5 Statistical analysis

Primary outcome measures were NCT and SCT immediately post-stimulation with adjustment for baseline (estimating change in NCT and SCT). Complete case analysis was used to allow for appropriate comparison between conditions. Separate analyses were run for the NCT and SCT outcome measures. To illustrate the response from baseline for each condition, a forest plot with mean (95% confidence interval) change from baseline was generated. This is for illustrative not inferential purposes (Bland and Altman, 2011). Mean differences and 95% confidence intervals for the change from baseline between each condition and the reference condition (25 Hz stimulation at left concha) were estimated using mixed-effects regression models. In the model, post-stimulation cough threshold was the dependent variable, stimulation condition was the independent variable and baseline cough threshold was a covariate. Participant ID was included as a random effect. 25 Hz stimulation at left concha was chosen as the reference condition because it is the most commonly used stimulation parameter for stroke rehabilitation (Thompson et al., 2021). Model residuals were plotted and visually assessed for homoskedasticity and normality. Statistical significance was set at *p* < 0.05. Statistical analysis was undertaken in Stata 17.0 (StataCorp, Texas).

## 3 Results

### 3.1 Participants

Sixteen healthy participants were recruited between March and August 2022 and completed the study. Four participants were excluded from NCT analysis—three of them did not have a C2 response to the highest concentration of citric acid in at least one session, and one participant did not attend the last visit due to family bereavement. Six participants were excluded from SCT analysis—five of them did not have a C2 response to the highest concentration of citric acid in at least one session, and one participant did not complete baseline SCT testing due to error in data collection.

### 3.2 Nebulizer output

The nebulizer had a mean (SD) output of 0.12 (0.005) mL/breath (0.12 × 3 = 0.36 mL per concentration) with a coefficient of variation of 4.45%. A coefficient of variation of <10% is considered adequately reliable (Crapo, 2000).

### 3.3 Safety and tolerability

Four participants reported mild side effects (ear sensitivity and ear pain during stimulation, and headache and coughing after stimulation). One participant reported moderate-to-severe ear pain during stimulation in one session, which was due to inadequate electrode gel placed on the electrodes; pain subsided completely when the electrode was re-adhered with an adequate amount of gel. All other side effects subsided within 1–2 days. Due to the long-term nature of the study (8 visits over several weeks/months), participants were not always certain whether side effects such as headache or coughing were due to stimulation or chronic illnesses, particularly if they occurred hours or days after the session.

### 3.4 Cough thresholds for each stimulation condition

**Figure 3:**
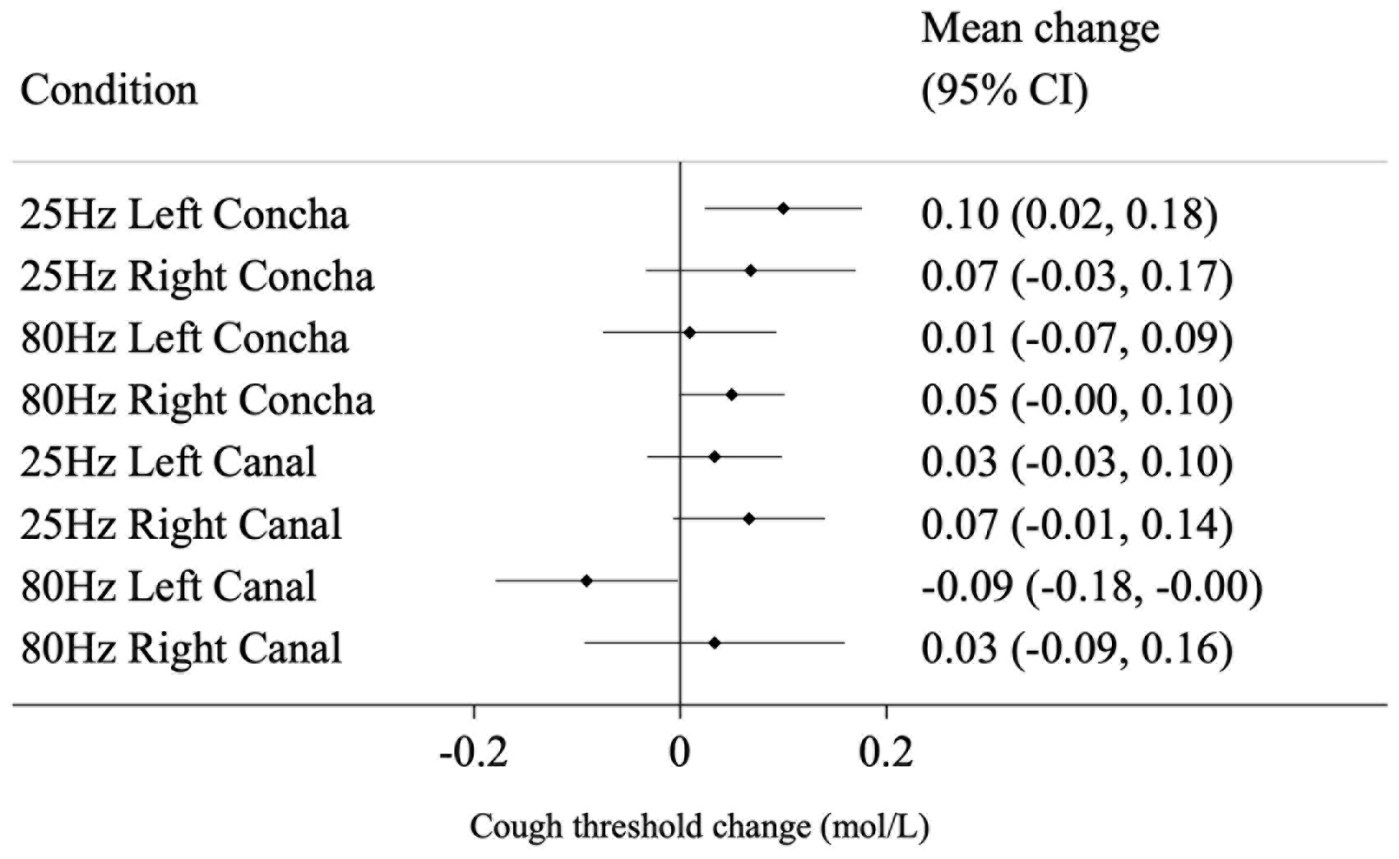
Mean change (from baseline to post-stimulation) in natural cough threshold for each condition, in mol/L. Horizontal lines represent 95% CI.

**Figure 4:**
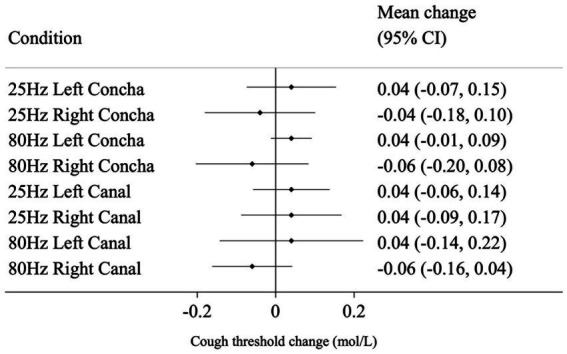
Mean change (from baseline to post-stimulation) in suppressed cough threshold for each condition, in mol/L. Horizontal lines represent 95% CI.

Mean and 95% CI change from baseline for all conditions are shown in Figure 3 for NCT, and Figure 4 for SCT. For the standard reference condition (25 Hz stimulation at left concha) NCT increased on average by 0.10 mol/L (95% CI: 0.02, 0.18). For 80 Hz stimulation at left canal, NCT had a reduction of 0.09 mol/L from baseline (95% CI: −0.18, 0.00), indicating an increase in cough sensitivity. These are shown to illustrate the responses to the different stimulation condition, but caution should be applied when interpreting pre-post tests like these (Bland and Altman, 2011).

### 3.5 Effect of stimulation condition on NCT and SCT

The mean difference in change from baseline between the reference condition (25 Hz stimulation at left concha) and each of the other seven conditions are presented in Table 1 and represent the estimated effect of different stimulation conditions compared to the reference. 80 Hz stimulation at the left canal had a statistically significant effect on NCT of −0.16 mol/L (*p* = 0.004) compared to the reference condition. For SCT, there was no evidence that the other seven conditions performed differently from the reference condition in changing cough threshold.

**Table 1:** Effect of condition on cough threshold, compared to reference parameter.

	Mean (SD) cough threshold (mol/L)			Mean (95% CI) difference in change from reference^
	Baseline	Post-stimulation	Change*	
**Natural cough test (n = 12)**				
25 Hz Left concha	0.18 (0.16)	0.28 (0.26)	0.10 (0.13)	Reference
25 Hz Right concha	0.17 (0.10)	0.24 (0.21)	0.07 (0.18)	−0.04 (−0.14, 0.07)
80 Hz Left concha	0.20 (0.14)	0.21 (0.17)	0.01 (0.15)	−0.09 (−0.19, 0.02)
80 Hz Right concha	0.22 (0.16)	0.27 (0.19)	0.05 (0.09)	−0.04 (−0.14, 0.07)
25 Hz Left canal	0.22 (0.20)	0.25 (0.23)	0.03 (0.12)	−0.06 (−0.16, 0.05)
25 Hz Right canal	0.22 (0.13)	0.28 (0.20)	0.07 (0.13)	−0.02 (−0.13, −0.08)
80 Hz Left canal	0.28 (0.20)	0.19 (0.12)	−0.09 (0.16)	−0.16 (−0.26, −0.05)
80 Hz Right canal	0.25 (0.21)	0.28 (0.20)	0.03 (0.22)	−0.04 (−0.15, 0.06)
**Suppressed cough test (n = 10)**				
25 Hz Left concha	0.32 (0.18)	0.36 (0.30)	0.04 (0.18)	Reference
25 Hz Right concha	0.42 (0.27)	0.38 (0.32)	−0.04 (0.23)	−0.04 (−0.19, 0.11)
80 Hz Left concha	0.30 (0.27)	0.34 (0.26)	0.04 (0.08)	−0.01 (−0.16, 0.14)
80 Hz Right concha	0.42 (0.25)	0.36 (0.25)	−0.06 (0.23)	−0.06 (−0.21, 0.09)
25 Hz Left canal	0.46 (0.35)	0.5 (0.39)	0.04 (0.16)	0.05 (−0.11, 0.21)
25 Hz Right canal	0.32 (0.24)	0.36 (0.27)	0.04 (0.21)	0.00 (−0.15, 0.15)
80 Hz Left canal	0.36 (0.14)	0.4 (0.36)	0.04 (0.30)	0.01 (−0.14, 0.17)
80 Hz Right canal	0.44 (0.28)	0.38 (0.19)	−0.06 (0.17)	−0.06 (−0.21, 0.10)

*Change in cough threshold is calculated as the difference between baseline and post-stimulation.

^The mean difference in change from reference is an estimated effect of condition using the regression model.

## 4 Discussion

This study evaluated the effect of various taVNS parameters on cough reflex threshold, compared to the reference parameter of 25 Hz stimulation at the left concha, commonly used in stroke rehabilitation. We found that 80 Hz stimulation at the left canal had a statistically significant but small reduction in natural cough threshold (indicating an increase in cough sensitivity) compared to the reference parameter. It is important to note that an active control was used in this study, and therefore interpretation of the results is in relation to the reference condition. Results from this study may guide future research decisions, as protocols investigating taVNS for sensitization of cough after stroke might select the parameters of 80 Hz stimulation at the left canal instead of the more commonly used parameters of 25 Hz stimulation at the left concha.

While up-regulation of cough sensitivity may be targeted for stroke patients with hyposensitivity, patients with laryngeal hypersensitivity such as those with chronic cough or obstructive sleep apnea (Shi et al., 2019) would benefit from down-regulation. Cough hypersensitivity involves the abnormally heightened response to laryngeal stimuli resulting in excessive coughing (Sundar et al., 2021). In our study, 25 Hz stimulation at the left concha resulted in an increase in natural cough threshold compared to 80 Hz stimulation at the left canal, and therefore 25 Hz taVNS at the left concha should be further investigated for de-sensitization of cough. Although there was a significant difference between 80 Hz stimulation at the left canal and 25 Hz stimulation at the left concha, caution should be applied when interpreting pre-post effects of each stimulation condition on its own, due to the lack of control group. Future work should incorporate the use of sham stimulation to better elucidate the effect of any one taVNS parameter on cough sensitivity.

The parameters of 25 Hz stimulation at the left concha is widely used in non-invasive taVNS studies for limb rehabilitation because these parameters have carried over from the invasive VNS domain (Thompson et al., 2021). However, it is important to have rationale for the specific parameters chosen for specific populations and specific impairments, as we know that outcomes are greatly influenced by the parameters used. If planning a stroke trial for limb rehabilitation, researchers should think carefully about choosing the commonly used 25 Hz concha parameter, and might consider choosing 80 Hz canal stimulation to avoid the risk of further dampening cough sensitivity. For stroke patients who may already have reduced or absent cough reflex due to neurological effects of stroke, 25 Hz stimulation on the left concha may result in unintended adverse effects on cough sensitivity. On the other hand, the effect of 80 Hz canal stimulation on limb function after stroke is unknown and should be further studied. Stimulation parameters should be appropriately tailored to target the specific physiological impairment.

Our findings suggest that higher frequency stimulation at the ear canal reduces cough thresholds compared to lower frequency stimulation at the concha. Previous research has indicated that high frequency taVNS (at least 80 Hz) was better than low (10–30 Hz) frequencies at improving smell function (Maharjan et al., 2018), modulating heart rate (Yokota et al., 2022), and reducing seizure activity (Jiao et al., 2016), possibly due to having a higher energy density (Badran et al., 2018). For implanted VNS, higher frequencies result in stronger but shorter duration of cortical activity compared to lower frequencies (Hulsey et al., 2017). An fMRI study demonstrated that high frequency taVNS was effective at eliciting increased brainstem activity in the medulla compared to low frequency stimulation, suggesting frequency-dependent cortical effects of taVNS (Sclocco et al., 2020). The medulla (including areas such as the paratrigeminal nucleus) plays a key role in integrating vagal afferent input and mediating the cough response (Bautista et al., 2019; Driessen, 2019; Mazzone et al., 2020). In this study, it is hypothesized that the increased energy of high-frequency stimulation led to increased afferent activation of the medulla and higher cortical structures that modulate cough, with the resulting up-regulation of cough sensitivity.

It is interesting to note that reduction in cough threshold was achieved with 80 Hz stimulation at the canal but not the concha, suggesting that location of stimulation can dictate outcomes and that the ear canal is a promising target for cough rehabilitation. This result appears to contradict a fMRI study revealing that taVNS at the ear canal resulted in lesser activation of the medulla compared to stimulation at the concha (Yakunina et al., 2017). The different outcomes could be explained by the different stimulation methods: the Yakunina et al. (2017) study targeted only the inferoposterior wall of the ear canal, while our study stimulated both the anterior and posterior walls of the ear canal and thus could have activated a larger number of vagal nerve fibers in the ear canal. The ear canal may have been a more effective target than the concha in our study because a previous neuroanatomical study demonstrated the higher density of nerve fibers in the ear canal compared to the concha (Bermejo et al., 2017). The potential for the ear canal being used for cough sensitization has been noted in several previous studies, where mechanical stimulation of the auricular nerve at the outer ear canal using capsaicin ointment increased cough reflex sensitivity and swallowing function (Kondo et al., 2014, 2017; Jinnouchi et al., 2020; Ohnishi et al., 2020). Further investigation should be completed to elucidate the mechanisms underlying parameter-specific effects of taVNS.

Although 80 Hz stimulation at the left canal was statistically different from 25 Hz stimulation at the left concha, the magnitude of change (0.16 mol/L) was relatively small, considering the cough reflex test delivered citric acid in increments of 0.2 mol/L. In addition, only one NCT condition and none of the SCT conditions were significantly different from the reference parameter. One potential explanation for this is that the stimulation duration of the current protocol (one 10-min session of each condition) was relatively brief. Principles of neuroplasticity state that sensitization protocols should be repetitive and intensive, so that a certain threshold of sensory input is reached to induce behavioral and cortical changes (Kleim and Jones, 2008; Dinse and Tegenthoff, 2015). While the optimal duration of treatment is unknown, and there is no common duration across studies, a previous taVNS protocol for sensory rehabilitation after stroke delivered 60-min sessions, 3 times a week for 6 weeks (Baig et al., 2019). Treatment parameters should be manipulated (e.g., increase duration and number of stimulation sessions) in future studies to maximize any potential treatment effects.

### 4.1 Limitations

There are several limitations of this study to address. As previously mentioned, there was no passive control group, making it difficult to assess the effect of stimulation by comparing active stimulation with no or sham stimulation. Cough threshold may change upon repeated testing after 10 min even without stimulation, and therefore it is unknown whether changes in cough threshold truly reflect the effect of stimulation. It is possible that cough threshold increases over time when people are exposed to citric acid cough testing multiple times in one day (Morice et al., 1992; Wallace et al., 2019).

Additionally, approximately one-third of participants did not respond with SCT at the highest concentration of citric acid, and hence were not included in the complete case analysis. This is in line with previous studies, where 21–32% of healthy participants do not respond with SCT at the highest concentration of citric acid during cough testing (Monroe et al., 2014; Mills et al., 2017; Perry and Huckabee, 2017). It is unknown how the cough sensitivity of these non-responders would have changed with stimulation if we were able to capture their true SCT. More work needs to be done developing valid and reliable measures of oral, pharyngeal, and laryngeal sensitivity that capture change over time.

## 5 Conclusion

One brief session of 80 Hz taVNS at the left canal resulted in a small increase in cough sensitivity when compared to the effect of 25 Hz stimulation at the left concha, suggesting that 80 Hz stimulation at the left canal should be considered instead of 25 Hz stimulation at the left concha for stroke patients with absent cough. Future trials should be conducted in healthy and neurological populations to investigate the potential modulatory effect of these two stimulation parameters on cough sensitivity, using a control group to elucidate the effects of each condition and increased treatment duration to maximize any potential treatment effects.

## Data availability statement

The datasets presented in this article are not readily available because ethics application and approval do not comprise data sharing.

## Ethics statement

The studies involving humans were approved by University of Otago Human Ethics (Health) Committee, reference (number H21/106). The studies were conducted in accordance with the local legislation and institutional requirements. The participants provided their written informed consent to participate in this study.

## Author contributions

KN: Conceptualization, Funding acquisition, Methodology, Project administration, Writing – review & editing, Validation, Data curation, Formal analysis, Investigation, Writing – original draft. EGH: Conceptualization, Formal analysis, Methodology, Validation, Writing – review & editing. JH: Validation, Writing – review & editing, Formal analysis. PM: Conceptualization, Funding acquisition, Methodology, Resources, Supervision, Validation, Writing – review & editing. M-LH: Conceptualization, Methodology, Validation, Writing – review & editing, Funding acquisition, Project administration, Resources, Supervision. YC: Conceptualization, Funding acquisition, Methodology, Project administration, Resources, Supervision, Writing – review & editing.
